# Blood Orange (*Citrus sinensis* L. Osbeck) Juice By-Product Extract as a Functional Feed Additive: Effects on Growth Performance, Digestive Enzyme Activity, Antioxidant Status, Immune Parameters, and Disease Resistance Against *Vibrio harveyi* in Juvenile Black Rockfish (*Sebastes schlegelii*)

**DOI:** 10.3390/antiox14060745

**Published:** 2025-06-17

**Authors:** Ahyeong Yun, Hwa Yong Oh, Tae Hoon Lee, Da Ye Kang, Ki-Tae Kim, Hyun-Soo Kim, Hee Sung Kim

**Affiliations:** 1 Ocean Science and Technology School, National Korea Maritime and Ocean University, Busan 49112, Republic of Korea; yoy0728@naver.com; 2 Department of Marine Biology and Aquaculture, Gyeongsang National University, Tongyeong 53064, Republic of Korea; oho1203@gnu.ac.kr (H.Y.O.); dlxogns1204@naver.com (T.H.L.); honey3292@naver.com (D.Y.K.); 3 Southeast Sea Fisheries Research Institute, National Institute of Fisheries Science, Tongyeong 53017, Republic of Korea; oysterkim@korea.kr; 4 Department of Seafood Science and Technology, Gyeongsang National University, Tongyeong 53064, Republic of Korea; gustn783@gnu.ac.kr

**Keywords:** *Citrus sinensis*, by-products, growth performance, health status, disease resistance, *Sebastes schlegelii*

## Abstract

This study evaluated the antibacterial activity and residual functional compounds of blood orange (*Citrus sinensis* L. Osbeck) juice by-product extract (BJBE). The effects of dietary BJBE on growth performance, digestive enzyme activity, antioxidant status, immune parameters, and disease resistance against *Vibrio harveyi* were examined in juvenile black rockfish (*Sebastes schlegelii*). In total, 630 juvenile rockfish were randomly assigned to 21 rectangular tanks (50 L) for a feeding trial, with 30 fish per tank in triplicate. Seven isonitrogenous and isolipidic experimental diets were formulated with BJBE at 0 (control, BJBE0), 0.1 (BJBE0.1), 0.2 (BJBE0.2), 0.3 (BJBE0.3), 0.5 (BJBE0.5), 0.7 (BJBE0.7), and 1.0 (BJBE1) g kg^−1^. A disk diffusion assay confirmed BJBE’s strong antibacterial efficacy against *V*. *harveyi*. After an 8-week feeding trial, fish fed BJBE0.7 and BJBE1 exhibited significantly a greater final weight, weight gain, and specific growth rate compared with those fed BJBE0. Feed efficiency was significantly higher in fish fed BJBE0.7 than in those fed BJBE0. The protein efficiency ratio was significantly higher in fish fed BJBE0.3, BJBE0.5, BJBE0.7, and BJBE1 relative to those fed BJBE0. Intestinal amylase activity was significantly higher in fish fed BJBE0.7 and BJBE1 compared with those fed BJBE0, and trypsin activity was significantly higher in BJBE0.7-fed fish than in BJBE0-fed fish. In comparison to the BJBE0 diet, the plasma superoxide dismutase, catalase, and glutathione levels of fish fed BJBE0.7 and BJBE1 diets were significantly higher. Lysozyme activity and immunoglobulin M level in fish fed BJBE0.7 and BJBE1 were significantly higher than that in fish fed BJBE0. After a challenge with *V*. *harveyi*, disease resistance was significantly higher in fish fed BJBE0.5, BJBE0.7, and BJBE1 compared with those fed BJBE0. Overall, 0.7–1.0 g kg^−1^ is proposed as the optimal dietary BJBE inclusion level for enhancing growth performance, digestive enzyme activity, antioxidant status, immune parameters, and disease resistance against *V*. *harveyi* infection in juvenile black rockfish.

## 1. Introduction

In recent years, the concept of a circular economy, which enhances economic efficiency and environmental sustainability through waste recycling, has gained global attention [1]. The international community has also recognized this concept as an important agenda, and the Sustainable Development Goals proposed by the UN emphasize the need to ensure food security and promote sustainable agriculture and fishery development. Aquaculture, one of the fastest-growing food production industries, is considered a sustainable approach to diversifying the global food supply [2,3,4]. A promising strategy for improving aquaculture sustainability is the use of by-products as feed ingredients to improve resource efficiency.

Agricultural by-products, derived from the cultivation and processing of agricultural products, include fruit, vegetables, meat, poultry, dairy products, and crops [5], and they pose serious management challenges from both economic and ecological perspectives [6]. Among these, fruit by-products are a major contributor to municipal solid waste, causing an ongoing environmental issue [7]. These fruit by-products, composed of seeds, peels, pulp residues, and stems left over from the juice extraction process, are typically discarded [8]. However, research has shown that many fruit processing by-products contain bioactive compounds, including carbohydrates (such as pectin, cellulose, and hemicellulose), as well as secondary metabolites (e.g., phenolic compounds, alkaloids, glycosides, volatile oils, and mucilage), proteins, and lipids [6,9]. Although managing fruit by-products poses environmental challenges, they also represent a source of bioactive compounds that could benefit aquatic animals [10]. Thus, recycling these by-products as animal feed ingredients could contribute to resource efficiency and environmental conservation. Prior studies have explored the potential of various fruit by-products, including apple (*Malus domestica*) [11], banana (*Musa acuminata*) [12], citrus [13], grape (*Vitis vinfera*) [14], and pomegranate (*Punica granatum*) [15], as feed additives.

Blood orange (*Citrus sinensis* L. Osbeck), a citrus fruit originating in China centuries ago, has been cultivated in Sicily, Italy, since the 15th century [16]. Global blood orange production has reached around 79 million tons [17], with two primary varieties [18]: Spain’s ‘Sanguinelli’ and Italy’s ‘Tarocco’ and ‘Moro’, which are the most common varieties in the Mediterranean basin [19]. Blood oranges are mainly processed into juice and jam, generating substantial by-products, including peel and seeds, which can account for up to 50% of the raw fruit [20]. By-products from the *Citrus* genus, including blood oranges, contain bioactive compounds, such as polyphenols, flavonoids, limonin, and triterpenoids. To prevent accumulation and environmental pollution, these by-products are commonly repurposed as animal feed [21,22]. Studies have reported the benefits of citrus by-products, such as orange peel [23] and dehydrated lemon (*Citrus limon*) peel [24] in gilthead seabream (*Sparus aurata*), as ecofriendly feed additives for improving fish growth and health.

Due to the various benefits provided by the bioactive compounds present in plant by-products, research on the utilization of plant by-product extracts in aquaculture has been conducted [25]. Compared with raw materials, plant by-product extracts provide concentrated bioactive compounds [26,27]. These compounds exhibit antioxidant [26], antibacterial, and antiviral properties [28] and are expected to support fish health. Accordingly, various studies have examined the effects of plant by-product extracts on fish overall health and performance, including tomato paste by-product extract [29], grapefruit (*C*. *paradisi*) peel extract [30], and olive (*Olea europaea Leecino*) leaf extract [31]. However, research on blood orange juice by-product extract (BJBE) remains limited.

Black rockfish (*Sebastes schlegelii*), a carnivorous and commonly found fish in the Republic of Korea, China, and Japan, is a highly valued species in cage-based aquaculture in the Republic of Korea because its rapid growth, high market demand, and superior flesh quality [32]. In 2024, black rockfish culture production in the Republic of Korea reached 14,513 tons [33], comprising approximately 18% of the total aquaculture output. However, black rockfish aquaculture faces major losses due to bacterial infections caused by *Vibrio* spp. (Gram-negative), *Photobacterium* spp. (Gram-negative), and *Streptococcus* spp. (Gram-positive) [34]. Consequently, there is growing interest in sustainable, ecofriendly, cost-effective, and natural feed additives for improved disease prevention, productivity, and health in black rockfish, prompting numerous studies in this field. Previous research has evaluated plant-derived by-products, such as yacon (*Smallanthus sonchifolius*) [35], garlic (*Allium sativum*) [36], and ginger (*Zingiber officinale*) [37] juice by-products, as well as blood orange peel [22], as dietary supplements in juvenile black rockfish. However, no studies have assessed the effects of plant-derived by-product extracts at varying inclusion levels in juvenile black rockfish diets. Therefore, this study aimed to assess the antibacterial activity and functional compound content of BJBE and determine its optimal dietary inclusion level for enhancing growth performance, digestive enzyme activity, antioxidant status, immune parameters, and disease resistance against *V*. *harveyi* in juvenile black rockfish.

## 2. Materials and Methods

### 2.1. Preparation of BJBE

Fresh ‘Moro’ blood oranges were purchased from a local fruit store in Seogwipo-si, Jeju-do, Republic of Korea, and transferred to the Marine Bio-Education and Research Center, Gyeongsang National University (Tongyeong, Gyeongsangnam-do, Republic of Korea). The oranges were rinsed with water, air-dried at room temperature, and processed using a juice extractor (H-300L-DBFC03, Hurom Co., Ltd., Seoul, Republic of Korea), leaving behind the by-products. These were dried at 20 °C for 72 h using an agricultural product dryer (KED-M07D1, Kiturami Co., Ltd., Seoul, Republic of Korea) and then ground using a kitchen blender. To extract BJBE, 20 g of the powdered sample was mixed with 200 mL of 80% ethanol and stirred at 60 °C for 2 h. The mixture was filtered using a vacuum funnel, and the filtrate was mixed again with 200 mL of ethanol, stirred at 60 °C for 2 h, and filtered using the vacuum funnel. The final filtrate was stored in a refrigerator. Subsequently, the mixture was concentrated at 50 °C using a rotary evaporator (Eyela N-1300V-W, Tokyo Rikakikai Co., Ltd., Tokyo, Japan), and the resulting BJBE was stored at 4 °C prior to chemical and antibacterial activity analyses.

### 2.2. Chemical and Antibacterial Activity Analyses of BJBE

To analyze the vitamin C (VC) concentration in BJBE, the mobile phase consisted of a 0.05 M KH_2_PO_4_ solution (pH 2.8), with a flow rate of 1.0 mL min^−1^. Samples were manually homogenized in 10% cooling metaphosphoric acid, followed by centrifugation at 3000× *g* for 20 min. The supernatants were filtered using a 0.45 μm pore syringe filter (Sartorius, Gottingen, Germany) before HPLC analysis. The analysis was performed using a high-performance liquid chromatograph (HPLC; Agilent 1200 Series HPLC; Agilent Technologies, Anaheim, CA, USA) equipped with a C18 column (Symmetry^®^ 4.6 mm × 280 mm, particle size 5 μm; Waters, Milford, MA, USA) and an ultraviolet detector set at 254 nm.

The total phenolic content of BJBE was determined using the Folin–Ciocalteu reagent [38]. A 1 mL aliquot of this reagent was mixed with 50 µL of the sample and vortexed. After a 3 min reaction, 1 mL of 10% sodium carbonate solution was added. Following a 60 min incubation at room temperature, absorbance was measured at 700 nm. Gallic acid (Sigma-Aldrich Co., St Louis, MO, USA) was used as the standard.

Flavonoid content in BJBE was analyzed following an established method [39]. A 1 mL sample was diluted with 4.3 mL of 80% ethanol, followed by the addition 0.1 mL of 10% aluminum nitrate and 0.1 mL of 1 mol L^−1^ aqueous potassium acetate. After incubation in the dark at room temperature for 40 min, absorbance was measured at 415 nm. Quercetin (Sigma-Aldrich Co., St. Louis, MO, USA) served as the standard.

The ABTS radical scavenging activity of BJBE was assessed using a method described previously [40]. In a light-free environment, 7 mM ABTS stock solution was prepared by dissolving ABTS (Sigma-Aldrich, USA) in distilled water and mixed with an equal volume of 2.4 mM potassium persulfate (Sigma-Aldrich, USA) in distilled water. The mixture was allowed to react for 16 h to generate ABTS radicals. The working solution was diluted with distilled water until an absorbance of 1.5 at 414 nm was reached. A 50 µL aliquot of the sample or ascorbic acid (positive control) was mixed with 100 µL of the working solution, and absorbance was measured at 414 nm after 5 min at room temperature. The ABTS radical scavenging activity was expressed as the percentage inhibition of absorbance relative to the control, calculated using the following equation:

$$\mathrm{Scavenging}\;\mathrm{activity}\;\left(\%\right)=\left(1-\frac{A_{\mathrm{sample}}}{A_{\mathrm{control}}}\right)\;\times \;100$$

DPPH scavenging activity was measured following an established method [41]. A 100 μL aliquot of 150 μM DPPH in methanol was mixed with 80 μL of the sample or ascorbic acid and left undisturbed at room temperature for 10 min. Absorbance was then measured at 525 nm using a microplate reader (SpectraMax^®^ M2/M2e, Sunnyvale, CA, USA). The DPPH radical scavenging activity was expressed as the percentage inhibition of absorbance relative to the control, calculated using the following equation:

$$\mathrm{Scavenging}\;\mathrm{activity}\;\left(\%\right)=\left(1-\frac{A_{\mathrm{sample}}}{A_{\mathrm{control}}}\right)\;\times \;100$$

For antimicrobial activity, the bacterial strain *V*. *harveyi* (FP8370), a known fish pathogen, was obtained from the Korean Culture Collection of Aquatic Microorganisms, National Institute of Fisheries Science, Busan, Republic of Korea, and stored at −70 °C. A 10 μL aliquot of *V*. *harveyi* culture suspension was inoculated onto Brain Heart Infusion Agar (BHIA) plates and incubated at 37 °C for 24 h. The cultured bacteria were suspended in 100 μL of 1× phosphate-buffered saline buffer and spread onto fresh BHIA plates. Disk papers (positive control, negative control, and BJBE) were placed on the plates and incubated at 27 °C. Tetracycline (antibiotic disk) was used as the positive control, and a disk containing 20 μL of distilled water served as the negative control. BJBE was added to the disks at concentrations of 160, 80, 40, and 20 μL. The inhibition zone (mm) was measured to assess BJBE’s ability to inhibit *V*. *harveyi*, and the results were compared with those from the antibiotic disks.

### 2.3. Formulation of Experimental Diets

Seven isonitrogenous and isolipidic experimental diets were formulated (Table 1), each containing different levels of BJBE: 0 (control, BJBE0), 0.1 (BJBE0.1), 0.2 (BJBE0.2), 0.3 (BJBE0.3), 0.5 (BJBE0.5), 0.7 (BJBE0.7), and 1.0 (BJBE1) g kg^−1^ diet. To ensure homogeneity, all dry feed ingredients were thoroughly mixed. Fish oil, soybean oil, and distilled water were then added to form a dough. In BJBE-containing diets, BJBE was incorporated in place of distilled water from the BJBE0 diet. The dough was processed into 3 mm diameter pellets using a chopper (3 mm diameter; SL Machinery, Incheon, Republic of Korea) and dried at 20 °C for 48 h using an agricultural product dryer (KED-M07D1, Kiturami Co., Ltd., Seoul, Republic of Korea). The experimental diets were then stored at −20 °C until use.

### 2.4. Feeding Trial Condition and Experimental Design

Juvenile black rockfish were obtained from a commercial hatchery located in Namhae-gun, Gyeongsangnam-do, Republic of Korea. They were then moved to the Marine Bio-Education and Research Center at Gyeongsang National University, Tongyeong, Gyeongsangnam-do, Republic of Korea. The fish were fed a commercial extruded pellet diet (Jeil Feed Co., Haman, Gyeongsangnam-do, Republic of Korea; 52% crude protein and 10% crude lipids) for 2 weeks to acclimate them to experimental conditions before the feeding trial.

After a 24 h fasting period, 630 juvenile rockfish (initial average weight: 1.4 g) were randomly assigned to 21 flow-through rectangular tanks (50 L volume and 2.7 L min^−1^ water flow rate). For 8 weeks, the seven experimental diets were fed twice daily (09:00 and 17:00), with three replicates per diet. Daily, the feed intake per tank was recorded, and feces were removed via siphoning. Each tank was aerated adequately, and average water temperature, salinity, and dissolved oxygen levels were recorded to be 20.9 ± 0.09 °C, 31.6 ± 0.12 psu, and 5.8 ± 0.07 mg L^−1^, respectively, based on daily measurements using a YSI Pro Plus multiparameter instrument (YSI Inc., Yellow Springs, OH, USA).

### 2.5. Growth Performance Parameters

When the 8-week feeding trial ended, all fish were fasted for 24 h and then anesthetized with 150 ppm tricaine methanesulfonate (MS-222; Sigma-Aldrich Co., St. Louis, MI, USA), and the final weight and number of surviving individuals in each tank were recorded. Growth performance parameters (growth and feed utilization) were calculated, considering the total length and body weight of the fish in each tank. In addition, the liver and intestines were removed and weighed to calculate the hepatosomatic index (HSI) and viscerosomatic index (VSI). These were calculated using the following formulas:

Survival (SR, %) = (number of fish at the end of the trial/number of fish at the beginning of the trial) × 100

Weight gain (WG, g fish^−1^) = final body weight − initial body weight

Specific growth rate (SGR, %/day) = [(ln final weight of fish − ln initial weight of fish)/days of feeding] × 100

Feed intake (FI, g fish^−1^) = total dry feed intake/fish

Feed efficiency (FE) = WG of fish/feed consumed

Protein efficiency ratio (PER) = WG of fish/protein consumed

Condition factor (CF) = (fish weight/total length^3^) × 100

HSI (%) = (liver weight/whole-body weight) × 100

VSI (%) = (viscera weight/whole-body weight) × 100

### 2.6. Blood Sampling for Analysis

In each tank, 10 fish were randomly selected and anesthetized, and blood samples were collected from the caudal vein using both heparin-coated syringes and noncoated syringes. Samples collected with heparin-coated syringes were centrifuged at 7000 rpm and 4 °C for 15 min to separate plasma for biochemical indices and antioxidant enzyme activity analyses. Blood samples collected with noncoated syringes were allowed to clot for 30 min before centrifugation at 3000× *g* for 5 min to separate serum for lysozyme activity, immunoglobulin M (IgM), and interleukin-1 (IL-1) analysis. All blood samples were stored at −80 °C until further analysis.

### 2.7. Digestive Enzyme Activity

Intestinal samples were collected from blood-sampled fish and homogenized in 10 volumes (*v*/*w*) of ice-cold 0.86% physiological saline using a Tissue Lyser II (QIAGEN, Venlo, Netherlands) in an ice bath. The homogenates were centrifuged at 13,000 rpm and 4 °C for 10 min, and the supernatants were used to assess the enzymatic activity of amylase (AB102523), trypsin (AB102531), and lipase (AB102524) using a commercial enzyme-linked immunosorbent assay (ELISA) kit (Abcam, Cambridge, UK).

### 2.8. Proximate Body Composition and Plasma Biochemical Analyses

At the end of the 8-week feeding trial, 10 fish were randomly selected from each tank and anesthetized with MS-222. For chemical composition analysis, whole fish samples were finely chopped and homogenized into a paste. Moisture, crude protein, crude lipid, and ash contents were determined according to the standard procedures of the Association of Official Analytical Chemists [42]. Crude protein content (N × 6.25) was measured using a KD310–A–1015 KjelROC Analyzer (OPSIS Liquid LINE, Furulund, Sweden) following the Kjeldahl digestion method. The crude lipid content was determined using the Soxhlet extraction method with a Soxtec extractor (ST 243 Soxtec™; FOSS, Hillerod, Denmark). Moisture content was assessed by drying samples in an oven at 105 °C for 24 h, and ash content was measured following incineration in a muffle furnace at 550 °C for 4 h.

Plasma biochemical parameters, including aspartate aminotransferase (AST), alanine aminotransferase (ALT), total cholesterol (TCHO), total protein (TP), and glucose (GLU), were analyzed using Fujifilm DRI-CHEM slides (Fujifilm, Tokyo, Japan) with an automated chemistry system (Fuji Dri-Chem NX500i; Fujifilm, Tokyo, Japan).

### 2.9. Antioxidant Enzyme Activity Analysis

The activities of antioxidant enzymes, including superoxide dismutase (SOD), catalase (CAT), and glutathione (GSH) contents, were assessed in plasma samples using commercial ELISA kits—the SOD assay kit (706006), CAT assay kit (707018), and GSH assay kit (703018) (Cayman’s Assay Kit, Cayman Chemical, Ann Arbor, MI)—following the manufacturer’s instructions. Absorbance readings were obtained using a spectrophotometer (Thermo Scientific Multiskan GO, Vantaa, Finland).

### 2.10. Immune Parameter Analysis

Serum lysozyme activity was determined using a turbidimetric assay, as described in a previous study [43]. Briefly, 100 μL of test serum was added to a 1.9 mL suspension of *Micrococcus lysodeikticus* (0.2 mg mL^−1^; Sigma-Aldrich Co., St. Louis, MO, USA) dissolved in 0.05 M sodium phosphate buffer (pH 6.2). The reaction was conducted at 25 °C, and absorbance was measured at 530 nm every 15 min over a 60 min period using a spectrophotometer (Thermo Fisher Scientific, Tewksbury, MA, USA). Lysozyme activity was expressed as the amount of enzyme required to cause a 0.001 min^−1^ decrease in absorbance. Serum IgM (MBS1609188) and IL-1 (MBS1601705) levels were quantified using commercial ELISA kits (Cusabio, Bio-tech Co., Ltd., Wuhan, China, and MyBioSource Inc., San Diego, CA, USA, respectively), according to the manufacturers’ instructions.

### 2.11. Challenge Test Against V. harveyi

Ten fish were randomly selected from each tank for the bacterial challenge test. The *V*. *harveyi* strain used in this study was provided by the Korean Culture Collection of Aquatic Microorganisms, National Institute of Fisheries Science, Busan, Republic of Korea. Each fish was intraperitoneally injected with 0.1 mL of *V*. *harveyi* culture suspension at a concentration of 7.9 × 10^6^ CFU mL^−1^. During the 4-day challenge period, water temperature and dissolved oxygen levels were maintained at 22.1 ± 0.18 °C and 7.0 ± 0.28 mg L^−1^ [mean ± standard error (SE)], respectively. Fish survival was monitored daily, and deceased individuals were removed every 6 h.

### 2.12. Statistical Analysis

Prior to statistical analysis, percentage data were arcsine-transformed to ensure normality. Results are presented as means ± SE. The homogeneity of variance among treatments was assessed using Levene’s test. A one-way analysis of variance followed by Tukey’s HSD test was performed to determine significant differences among groups (*p* < 0.05). To analyze fish survival during the 4-day post-challenge period, Kaplan–Meier survival curves were generated, and survival differences were evaluated using the log-rank and Wilcoxon tests. All statistical analyses were conducted using SPSS version 27.0 (SPSS Inc., based in Chicago, IL, USA).

## 3. Results

### 3.1. Chemical Composition and Antioxidant Activities of BJBE

The VC content of BJBE was 41.93 mg 100 g^−1^, and the total phenolic and total flavonoid contents were 28.7 gallic acid mg 100 g^−1^ and 15.3 quercetin mg g^−1^, respectively (Table 2). According to DPPH and ABTS radical scavenging assay results, BJBE effectively suppressed free radical production in a concentration-dependent manner (Table 2).

### 3.2. Antibacterial Effect of BJBE Against V. harveyi

The antibacterial activity of BJBE against *V*. *harveyi* is presented in Table 3. The highest inhibition zone diameter, 24 mm, was observed at concentrations of 40 and 160 µL disk^−1^, whereas the lowest inhibition zone diameter, 17 mm, was recorded at 20 µL disk^−1^. In comparison, the antibiotic tetracycline exhibited an inhibition zone diameter of 15 mm. These results indicate that BJBE exhibited strong antibacterial activity against *V*. *harveyi*.

### 3.3. Growth Performance and Feed Utilization

The effects of dietary BJBE on growth performance, feed utilization, and organosomatic indices are summarized in Table 4. Final weight, WG, and SGR were significantly higher in fish fed BJBE0.7 and BJBE1 diets compared with those fed the BJBE0 diet (*p* < 0.05). However, SR and FI did not differ significantly among the dietary groups (*p* > 0.05). FE was significantly higher in fish fed BJBE0.7 than in those fed BJBE0 (*p* < 0.05), whereas no significant differences were detected among the other treatments (*p* > 0.05). The PER was significantly higher in fish fed BJBE0.3, BJBE0.5, BJBE0.7, and BJBE1 compared with those fed BJBE0 (*p* < 0.05), whereas no significant differences were observed between the BJBE0 group and the BJBE0.1 or BJBE0.2 group (*p* > 0.05). The CF, VSI, and HSI did not show significant differences among the dietary groups (*p* > 0.05).

### 3.4. Digestive Enzyme Activities

The activities of intestinal digestive enzymes, including amylase, trypsin, and lipase, in juvenile black rockfish according to treatment are shown in Table 5. Amylase activity was significantly higher in fish fed BJBE0.7 and BJBE1 compared with those fed BJBE0 (*p* < 0.05) but did not differ significantly from that in fish fed BJBE0.1, BJBE0.2, BJBE0.3, and BJBE0.5 (*p* > 0.05). Trypsin activity was significantly higher in fish fed BJBE0.7 relative to those fed BJBE0, BJBE0.1, and BJBE0.2 (*p* < 0.05), whereas no significant differences were found among fish fed BJBE0.3, BJBE0.5, and BJBE1 (*p* > 0.05). Dietary BJBE supplementation had no significant effect on juvenile black rockfish lipase activity (*p* > 0.05).

### 3.5. Whole-Body Composition and Blood Biochemical Indices

The effects of dietary BJBE on whole-body composition and blood biochemical indices are shown in Table 6. Moisture, crude protein, crude lipid, and ash contents showed no significant differences among the dietary treatments (*p* > 0.05). Similarly, plasma concentrations of AST, ALT, GLU, TP, and TCHO were not significantly affected by dietary BJBE inclusion (*p* > 0.05).

### 3.6. Antioxidant Enzyme Activities

The plasma antioxidant enzyme activities of juvenile black rockfish by treatment are presented in Table 7. Plasma SOD and CAT activities were significantly higher in fish fed BJBE0.7 and BJBE1 relative to those fed BJBE0 and BJBE0.1 (*p* < 0.05). Plasma GSH levels were significantly higher in the BJBE0.7 and BJBE1 groups relative to all other dietary groups (*p* < 0.05).

### 3.7. Immune Parameters

The immune parameters of juvenile black rockfish according to treatment are shown in Table 8. Serum lysozyme activity was significantly higher in fish fed BJBE0.7 and BJBE1 compared with those fed BJBE0 (*p* < 0.05) but did not differ significantly from that of fish fed BJBE0.2, BJBE0.3, and BJBE0.5 (*p* > 0.05). Serum IgM levels were significantly higher in the BJBE0.7 and BJBE1 groups compared with the BJBE0 group (*p* < 0.05) but did not differ significantly from levels observed in the BJBE0.5 group (*p* > 0.05). No significant differences were observed in serum IL-1 levels among the dietary treatments (*p* > 0.05).

### 3.8. Challenge Test

**Figure 1:**
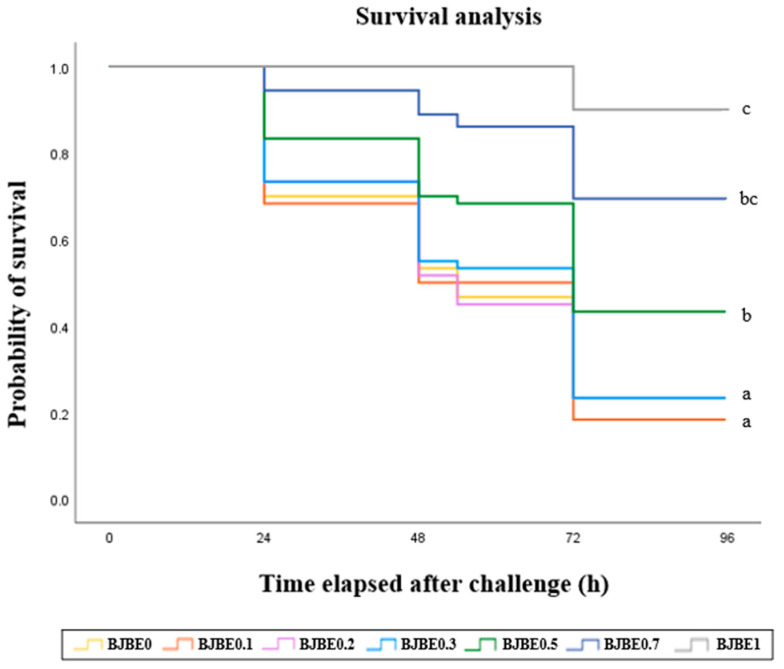
Survival rate of juvenile black rockfish fed experimental diets containing blood orange juice by-product extract (BJBE) for 8 weeks, followed by artificial infection with *Vibrio harveyi* (means of triplicate ± SE). Different letters next to the survival curve indicate significant differences among treatments at 96 h post-challenge (*p* < 0.001 for log-rank and Wilcoxon tests).

The survival of fish artificially infected with *V*. *harveyi* over 4 days post infection is shown in Figure 1. Fish fed BJBE0.5, BJBE0.7, and BJBE1 exhibited significantly higher survival rates compared with those fed BJBE0 (*p* < 0.05).

## 4. Discussion

The recycling of food production by-products as functional feed additives has gained increasing attention as a sustainable strategy to promote growth and enhance health in aquaculture. Several studies have highlighted the potential of these by-products as feed additives [44,45,46], showing their economic benefits, environmental advantages, and health-promoting effects in aquaculture species [47]. Therefore, this study aimed to analyze the functional compounds present in BJBE and evaluate their effects when incorporated into the diet of juvenile black rockfish, exploring BJBE’s potential as a novel functional and ecofriendly feed additive.

Citrus fruit are abundant sources of phytochemicals, such as VC, flavonoids, phenolic compounds, minerals, coumarins, limonoids, carotenoids, and pectins, among others [16,22,48]. These compounds exhibit a range of biological activities, including anticancer, anti-inflammatory, and antioxidant effects [49,50,51]. In particular, citrus extracts contain various phenolic acids and flavonoids, as well as high levels of ascorbic acid [52]. These components contribute to the extracts’ biological functions (e.g., antimicrobial and antioxidant effects), making them beneficial for aquatic species [53]. In this study, BJBE supplementation enhanced antioxidant and antimicrobial activities, likely due to the presence of key phytochemicals, such as VC, phenolic compounds, and flavonoids.

Notably, citrus essential oils have demonstrated antimicrobial, antioxidant, and biocidal effects against a range of fungi, viruses, and protozoa, suggesting that they can serve as effective microbial control agents [54,55]. The antibacterial properties of citrus essential oils have been confirmed against multiple pathogenic microorganisms, including *S*. *mutans*, *Lactobacillus acidophilus*, *Staphylococcus aureus*, and *Escherichia coli* [56]. In this study, BJBE supplementation inhibited *V*. *harveyi* growth. This antibacterial effect may be attributed to the presence of flavonoids, which are abundant in citrus extracts and have been widely reported to possess antibacterial properties [57]. Previous research has demonstrated that citrus extracts from grapefruit, mandarin (*C reticulata*), bergamot (*C*. *bergamia*), and sweet orange effectively inhibit growth and induce cell death in *Salmonella enterica*, *E*. *coli*, and *Brachyspira hyodysenteriae* [57]. In prior studies [58,59], citrus extracts were shown to contain various bioactive compounds, and their antibacterial effects were reported to result from a synergistic interaction among compounds rather than the action of a single active component. Similarly, the antibacterial activity of BJBE against *V*. *harveyi* observed in the current study is likely due to the combined action of multiple bioactive compounds, including flavonoids, on different targets in microbial cells, rather than a single mechanism. These findings suggest that BJBE not only inhibits the growth of specific pathogenic microorganisms but also potentially contributes to immune enhancement in juvenile black rockfish. Thus, BJBE holds promise as an antibacterial agent and a functional feed additive for improving fish immunity.

Growth enhancement is a critical factor in aquaculture, directly affecting profitability and productivity [60]. Additionally, optimizing FE is essential for reducing feed costs, which constitute a major proportion of total aquaculture expenses [61]. In the present study, dietary supplementation with 0.7 and 1.0 g kg^−1^ BJBE increased final body weight, WG, and SGR compared with the BJBE0 control group. Moreover, fish fed BJBE0.7 exhibited higher FE than fish fed BJBE0, and the PER was higher in fish receiving diets containing ≥0.3 g kg^−1^ BJBE compared with the BJBE0 group. These results align with previous findings [62] indicating that dietary supplementation with essential oils extracted from bitter lemon and sweet orange, rich in D-limonene, enhances growth performance and feed utilization in Nile tilapia. Similarly, the inclusion of grapefruit peel extract in Caspian white fish (*Rutilus frisii kutum*) diets resulted in improved growth performance and feed utilization [30]. The bioactive compounds found in orange by-products, including flavonoids, phenols, alkaloids, saponins, terpenes, resins, and tannins [63], have been shown to enhance growth performance and feed utilization by stimulating digestive enzyme activity, thereby improving nutrient digestion and absorption [64,65,66,67].

Digestive enzymes play a crucial role in nutrient digestion, directly influencing the digestive capacity and growth rate of fish [68]. Key enzymes, such as amylase, lipase, and trypsin (a type of protease) [69], markedly impact feed intake and growth performance [70]. However, their activity can vary depending on factors such as fish species, age, dietary enzyme composition, and feeding habits [71]. In the current study, the inclusion of dietary BJBE affected the activity of amylase and trypsin in juvenile black rockfish, whereas lipase activity remained unchanged. Similarly, a previous study reported that dietary *C*. *sinensis* peel extract enhanced amylase and protease activity in *Catla catla* [67], and dietary supplementation with lemon verbena (*Aloysia citrodora*) extract improved amylase activity in Siberian sturgeon (*Acipenser baerii*) [72]. Citrus peel, including sweet orange, is rich in phenolic compounds, known for their antimicrobial and antioxidant properties [73,74]. These compounds help regulate gut microbiota by increasing beneficial bacteria and suppressing pathogenic bacteria [75], ultimately enhancing nutrient absorption [76]. In the present study, the bioactive compounds in BJBE improved digestive enzyme activity, leading to better nutrient digestion and absorption, which contributed to the observed improvements in growth performance and feed utilization in juvenile black rockfish.

The proximate composition of fish serves as an important indicator of dietary nutrient efficacy and overall health status [67]. In the current study, moisture, protein, lipid, and ash contents were unaffected by dietary BJBE supplementation, suggesting that BJBE does not negatively affect the health of juvenile black rockfish. Similar findings have been reported in African catfish (*Clarias gariepinus*) fed rosemary (*Rosemaria officinalis*) extract [77]. Additionally, the whole-body composition of red hybrid tilapia (*Oreochromis mossambicus × O. niloticus*) was unaffected by different papaya (*Carica papaya*) leaf extract supplementation levels [78]. However, protein, lipid, and ash contents in the whole body of Nile tilapia were affected by dietary supplementation with pineapple waste crude extract [79]. These variations among studies may be attributed to differences in the species of additives used and their active compounds [80].

Blood biochemical indices provide valuable insights into fish health following different feeding trials [81]. In the present study, BJBE supplementation did not alter blood chemistry parameters in juvenile black rockfish, suggesting that it does not cause adverse health effects or liver toxicity. These results align with previous studies reporting that citrus by-products do not negatively impact hematological indices in fish [62,82]. However, the blood biochemical parameters of rainbow trout (*Oncorhynchus mykiss*) were affected by dietary lemon verbena (*Aloysia triphylla*) extract [83]. Such discrepancies may be attributed to differences in the plant species used in the feed additive [84], the plant parts utilized (e.g., roots, leaves, fruit, or seeds), and the processing and extraction methods employed [85].

The antioxidant system, primarily composed of SOD, CAT, and GSH, plays a crucial role in protecting living cells from free radicals, inflammation, and apoptosis [86,87]. These enzymes neutralize reactive oxygen species by converting superoxide molecules first into hydrogen peroxide and then into water [87]. In the current study, plasma SOD, CAT, and GSH levels were elevated in juvenile black rockfish fed diets supplemented with 0.7 and 1.0 g kg^−1^ BJBE. Similarly, a previous study has demonstrated increased antioxidant enzyme activity in rainbow trout supplemented with lemon balm extract [88]. Additionally, dietary inclusion of 10 g kg^−1^ blood orange peel powder enhanced antioxidant enzyme activity in juvenile black rockfish [22]. These findings may be due to the high antioxidant activity of various flavonoids, VC, and carotenoids present in citrus fruit [89]. Phenolic compounds, as secondary metabolites produced by plants [90], exhibit diverse biological properties, including antioxidant, antiaging, anticancer, anti-inflammatory, and cardioprotective effects, as well as the ability to inhibit angiogenesis and cell proliferation [91]. Moreover, flavonoids have been shown to modulate cellular responses to different stimuli by enhancing antioxidant defenses and activating intracellular signaling pathways that trigger antioxidant responses [92]. The observed increase in antioxidant enzyme activity in the present study is likely due to the presence of bioactive compounds in BJBE, particularly phenolic compounds (28.7 gallic acid mg 100 g^−1^) and flavonoids (15.3 quercetin mg g^−1^).

Lysozymes are key components of the innate immune system, functioning as antibacterial agents while enhancing phagocytosis through the direct stimulation of macrophages and polymorphonuclear leukocytes or via an opsonic effect [93]. IgM is the primary immunoglobulin in fish, playing a critical role in the initial immune response [94]. Additionally, IL-1 is a pro-inflammatory cytokine that acts early in the inflammatory response [95]. In this study, lysozyme activity and IgM levels were elevated in juvenile black rockfish fed diets containing 0.7 and 1.0 g kg^−1^ BJBE, and no significant differences were observed in those fed 0.5 g kg^−1^ BJBE. However, the IL-1 value remained unaffected by dietary BJBE, suggesting that its inclusion enhances the innate immune response by stimulating lysozyme activity and IgM production without inducing excessive inflammation. Similarly, dietary supplementation with Cornelian cherry (*Cornus mas*) extract increased lysozyme activity and total Ig levels in common carp (*Cyprinus carpio*) [96], and barberry fruit (*Berberis vulgaris*) extract enhanced lysozyme activity and IgM levels in Siberian sturgeon [97]. Conversely, dietary date palm (*Phoenix dactylifera*) extract did not alter IgM levels in European seabass (*Dicentrarchus labrax*) [98]. The inclusion of *Origanum* essential oil in common carp diets also increased lysozyme activity and the expression of interleukin-1β, a member of the IL-1 family [99]. Flavonoids, known for their strong immunostimulatory properties, are abundant in citrus fruit [100]. Therefore, the flavonoids present in BJBE likely contributed to the observed immune-enhancing effects. However, flavonoids are just one of many bioactive compounds in BJBE, making it difficult to attribute these effects solely to these components. A prior study [101] indicated that immune stimulation and anti-inflammatory responses depend on the type of additive used and the interactions among its various active compounds. Thus, the immune-enhancing effects of BJBE observed in the present study are likely due to the synergistic interactions among flavonoids and other bioactive compounds. Further research should focus on elucidating these interactions and conducting molecular analyses, including expression analysis of IL-1-related genes, to better understand BJBE’s immune-regulatory mechanisms.

Bacterial challenge tests are widely used to evaluate the protective effects of dietary interventions against pathogens, serving as key indicators of fish health [102]. In the present study, the survival rate of juvenile black rockfish challenged with *V*. *harveyi* was higher in those fed ≥0.5 g kg^−1^ BJBE compared with the BJBE0 group. This aligns with previous findings [103] demonstrating that plant-derived flavonoids improve immune mediator levels and enhance resistance to *Aeromonas hydrophila* infection. Similarly, dietary supplementation with sweet orange peel essential oil has been shown to increase tilapia (*O. mossambicus*) resistance to *S*. *iniae* infection [104]. Other studies have reported that dietary henna (*Lawsonia inermis*) [105] extracts enhance *A*. *hydrophila* resistance in common carp. The immunostimulatory properties of plant-derived bioactive compounds effectively reduce disease incidence in aquaculture by boosting immunity [106,107], further supporting the possibility that BJBE supplementation contributes to improved pathogen resistance in juvenile black rockfish. Notably, the positive effect of BJBE on *V*. *harveyi* resistance suggests its potential as an immunostimulant for enhancing fish health and disease resistance.

## 5. Conclusions

In conclusion, this study demonstrated that dietary BJBE supplementation positively influences growth performance, digestive enzyme activity, antioxidant status, immune parameters, and disease resistance against *V*. *harveyi* in juvenile black rockfish. The dietary inclusion of 0.7–1.0 g kg^−1^ BJBE as an ecofriendly immunostimulant and growth promoter holds promise for enhancing fish overall health and aquaculture productivity. However, this study was limited to a single species, and the effects of BJBE may vary depending on the fish species. To validate BJBE’s broader applicability, further studies across diverse species are warranted. Additionally, comprehensive assessments of long-term feeding effects, production cost, and application efficiency are necessary to evaluate its commercial potential.

## Figures and Tables

**Table 1:** Composition and proximate analysis of the experimental diets containing different levels of blood orange juice by-product extract (BJBE, expressed as g kg^−1^ dry matter).

Ingredients	Experimental Diets						
	BJBE0	BJBE0.1	BJBE0.2	BJBE0.3	BJBE0.5	BJBE0.7	BJBE1
Sardine meal	560	560	560	560	560	560	560
Dehulled soybean meal	120	120	120	120	120	120	120
Wheat flour	215	215	215	215	215	215	215
BJBE	0	0.1	0.2	0.3	0.5	0.7	1
Fish oil	40	40	40	40	40	40	40
Soybean oil	40	40	40	40	40	40	40
Vitamin premix ^a^	10	10	10	10	10	10	10
Mineral premix ^b^	10	10	10	10	10	10	10
Choline	5	5	5	5	5	5	5
*Proximate composition* (g kg^−1^)							
Dry matter	917	920	920	922	918	920	919
Crude protein	526	521	521	510	515	508	511
Crude lipid	145	148	148	145	145	139	145
Ash	105	104	105	98	104	102	104

^a^ Vitamin premix contained the following amounts of each ingredient, which were diluted in cellulose (g kg^−1^ mix): L-ascorbic acid, 121.2; DL-α-tocopheryl acetate, 18.8; thiamin hydrochloride, 2.7; riboflavin, 9.1; pyridoxine hydrochloride, 1.8; niacin, 36.4; Ca-D-pantothenate, 12.7; myo-inositol, 181.8; D-biotin, 0.27; folic acid, 0.68; p-aminobenzoic acid, 18.2; menadione, 1.8; retinyl acetate, 0.73; cholecalciferol, 0.003; cyanocobalamin, 0.003. ^b^ Mineral premix contained the following ingredients (g kg^−1^ mix): MgSO_4_·7H_2_O, 80.0; NaH_2_PO_4_·2H_2_O, 370.0; KCl, 130.0; ferric citrate, 40.0; ZnSO_4_·7H_2_O, 20.0; Ca-lactate, 356.5; CuCl, 0.2; AlCl_3_·6H_2_O, 0.15; KI, 0.15; Na_2_Se_2_O_3_, 0.01; MnSO_4_·H_2_O, 2.0; CoCl_2_·6H_2_O, 1.0.

**Table 2:** Vitamin C, total phenolic, and total flavonoid contents and radical scavenging activity of blood orange juice by-product extract (BJBE).

BJBE Composition								
Chemical compounds	Vitamin C (mg 100 g^−1^)			41.93				
	Total phenolics (gallic acid mg 100 g^−1^)			28.7 ± 3.48				
	Total flavonoids (quercetin mg g^−1^)			15.3 ± 7.37				
	Concentration (µg mL^−1^)	4000	2000	1000	500	250	125	IC50
Radical scavenging activity	DPPH (%)	61.2 ± 0.95	41.9 ± 2.14	33.4 ± 0.80	24.7 ± 0.61	22.3 ± 0.09	21.4 ± 0.06	5.5
	ABTS (%)	69.5 ± 0.95	35.5 ± 4.59	31.0 ± 0.35	19.9 ± 0.30	12.9 ± 0.04	6.2 ± 0.04	5.5

Values are means ± SE (n = 3). Abbreviations: DPPH, 1,1–diphenyl–2–picrylhydrazyl; ABTS, 2,2′–azinobis—(3–ethylbenzothiazoline–6–sulfonate).

**Table 3:** Antibacterial activity of blood orange juice by-product extract (BJBE) and an antibiotic against *Vibrio harveyi*, assessed using an agar disk diffusion assay.

Extracts/Antibiotic	Antibacterial Activity of Extracts	
	Concentration (μL disk^−1^)	Inhibition Zone Diameter (mm)
Tetracycline (30 µg)	-	1.5
BJBE	20	1.7
	40	2.4
	80	2.0
	160	2.4

**Table 4:** Growth performance and feed utilization of juvenile black rockfish fed experimental diets containing different levels of blood orange juice by-product extract (BJBE) for 8 weeks.

Parameters	Experimental Diets							*p* Value
	BJBE0	BJBE0.1	BJBE0.2	BJBE0.3	BJBE0.5	BJBE0.7	BJBE1	
Initial weight (g fish^−1^)	1.4 ± 0.00	1.4 ± 0.00	1.4 ± 0.00	1.4 ± 0.00	1.4 ± 0.00	1.4 ± 0.00	1.4 ± 0.00	-
Final weight (g fish^−1^)	10.2 ± 0.24 ^a^	10.5 ± 0.08 ^ab^	10.5 ± 0.12 ^ab^	10.7 ± 0.18 ^ab^	10.7 ± 0.11 ^ab^	11.0 ± 0.14 ^b^	10.9 ± 0.12 ^b^	0.031
SR (%)	100.0 ± 0.00	98.9 ± 1.11	100.0 ± 0.00	100.0 ± 0.00	100.0 ± 0.00	100.0 ± 0.00	100.0 ± 0.00	0.463
WG (g fish^−1^)	8.8 ± 0.24 ^a^	9.2 ± 0.08 ^ab^	9.2 ± 0.12 ^ab^	9.3 ± 0.18 ^ab^	9.3 ± 0.11 ^ab^	9.6 ± 0.14 ^b^	9.6 ± 0.12 ^b^	0.029
SGR (%)	3.66 ± 0.044 ^a^	3.73 ± 0.014 ^ab^	3.72 ± 0.024 ^ab^	3.75 ± 0.029 ^ab^	3.75 ± 0.017 ^ab^	3.80 ± 0.026 ^b^	3.79 ± 0.020 ^b^	0.039
FI (g/fish)	10.0 ± 0.04	10.1 ± 0.08	10.2 ± 0.04	10.0 ± 0.09	9.9 ± 0.13	10.1 ± 0.13	10.1 ± 0.06	0.463
FE	0.88 ± 0.021 ^a^	0.90 ± 0.009 ^ab^	0.90 ± 0.013 ^ab^	0.94 ± 0.014 ^ab^	0.94 ± 0.018 ^ab^	0.95 ± 0.014 ^b^	0.95 ± 0.007 ^ab^	0.022
PER	1.68 ± 0.040 ^a^	1.73 ± 0.017 ^ab^	1.73 ± 0.026 ^ab^	1.84 ± 0.027 ^bc^	1.83 ± 0.035 ^bc^	1.88 ± 0.027 ^c^	1.85 ± 0.014 ^bc^	0.001
CF	0.79 ± 0.040	0.87 ± 0.075	0.83 ± 0.046	0.84 ± 0.036	0.87 ± 0.058	0.85 ± 0.030	0.85 ± 0.060	0.924
VSI (%)	14.94 ± 0.037	14.83 ± 0.339	14.48 ± 0.246	14.38 ± 0.276	14.42 ± 0.239	14.20 ± 0.219	14.35 ± 0.187	0.329
HSI (%)	4.88 ± 0.087	4.59 ± 0.098	4.75 ± 0.255	4.56 ± 0.150	4.62 ± 0.200	4.85 ± 0.065	4.81 ± 0.200	0.696

Values are means ± SE (n = 3). Values with different superscript letters within a row indicate significant differences (*p* < 0.05), whereas values in the same row without superscript letters are not significantly different. Abbreviations: SR, survival; WG, weight gain; SGR, specific growth rate; FI, feed intake; FE, feed efficiency; PER, protein efficiency ratio; CF, condition factor; VSI, viscerosomatic index; HSI, hepatosomatic index.

**Table 5:** Digestive enzyme activities (mU mg^−1^ protein) in juvenile black rockfish fed experimental diets containing different levels of blood orange juice by-product extract (BJBE) for 8 weeks.

Parameters	Experimental Diets							*p* Value
	BJBE0	BJBE0.1	BJBE0.2	BJBE0.3	BJBE0.5	BJBE0.7	BJBE1	
Amylase	72.2 ± 7.83 ^a^	80.6 ± 7.38 ^ab^	88.4 ± 5.44 ^ab^	89.4 ± 5.24 ^ab^	104.6 ± 4.27 ^ab^	107.0 ± 4.19 ^b^	106.8 ± 10.76 ^b^	0.014
Trypsin	35.6 ± 0.74 ^a^	35.4 ± 0.52 ^a^	36.0 ± 1.83 ^a^	36.6 ± 1.96 ^ab^	37.5 ± 1.40 ^ab^	42.6 ± 1.50 ^b^	41.9 ± 0.72 ^ab^	0.006
Lipase	32.4 ± 4.52	32.5 ± 2.79	31.7 ± 1.73	32.2 ± 0.40	32.0 ± 1.38	33.2 ± 2.01	33.4 ± 1.79	0.998

Values are means ± SE (n = 3). Values with different superscript letters within a row indicate significant differences (*p* < 0.05), whereas values in the same row without superscript letters are not significantly different.

**Table 6:** Proximate composition (%) and blood biochemical parameters of juvenile black rockfish fed experimental diets containing different levels of blood orange juice by-product extract (BJBE) for 8 weeks.

Composition	Experimental Diets							*p* Value
	BJBE0	BJBE0.1	BJBE0.2	BJBE0.3	BJBE0.5	BJBE0.7	BJBE1	
Moisture	69.4 ± 0.15	69.3 ± 0.07	69.4 ± 0.15	69.4 ± 0.19	69.4 ± 0.25	69.5 ± 0.12	69.3 ± 0.22	0.996
Crude protein	17.0 ± 0.20	16.5 ± 0.03	16.5 ± 0.07	16.8 ± 0.07	16.5 ± 0.09	16.8 ± 0.24	16.7 ± 0.09	0.090
Crude lipid	9.6 ± 0.10	9.5 ± 0.12	9.2 ± 0.10	9.6 ± 0.06	9.4 ± 0.09	9.4 ± 0.00	9.4 ± 0.09	0.072
Ash	3.2 ± 0.09	3.3 ± 0.06	3.3 ± 0.12	3.4 ± 0.09	3.3 ± 0.15	3.1 ± 0.03	3.2 ± 0.07	0.460
AST (U L^−1^)	153.0 ± 4.58	153.3 ± 4.63	157.3 ± 7.17	153.7 ± 5.36	151.0 ± 6.24	153.3 ± 6.36	153.0 ± 7.23	0.995
ALT (U L^−1^)	40.0 ± 4.93	41.0 ± 5.57	40.7 ± 7.22	43.0 ± 6.66	45.0 ± 7.23	44.7 ± 4.91	44.3 ± 5.61	0.993
TCHO (mg dL^−1^)	242.3 ± 11.55	247.3 ± 12.78	242.7 ± 11.29	238.3 ± 7.31	244.0 ± 11.93	245.0 ± 10.21	245.3 ± 13.37	0.999
GLU (mg dL^−1^)	63.7 ± 8.11	62.3 ± 7.31	63.0 ± 5.57	63.3 ± 4.33	67.3 ± 4.91	65.7 ± 4.10	65.0 ± 5.29	0.996
TP (g dL^−1^)	5.6 ± 0.26	5.7 ± 0.61	6.3 ± 0.75	5.6 ± 0.41	5.7 ± 1.14	6.3 ± 1.03	5.5 ± 0.35	0.960

Values are means ± SE (n = 3). Values are presented without superscript letters, indicating no significant differences within rows (*p* > 0.05), whereas values in the same row without superscript letters are not significantly different. Abbreviations: AST, aspartate aminotransferase; ALT, alanine aminotransferase; TCHO, total cholesterol; GLU, glucose; TP, total protein.

**Table 7:** Plasma antioxidant parameters in juvenile black rockfish fed experimental diets containing different levels of blood orange juice by-product extract (BJBE) for 8 weeks.

Parameters	Experimental Diets							*p* Value
	BJBE0	BJBE0.1	BJBE0.2	BJBE0.3	BJBE0.5	BJBE0.7	BJBE1	
SOD (U mL^−1^)	7.1 ± 0.28 ^a^	7.4 ± 0.47 ^a^	7.7 ± 0.37 ^ab^	7.8 ± 0.23 ^ab^	8.1 ± 0.24 ^ab^	8.6 ± 0.34 ^b^	8.7 ± 0.32 ^b^	0.033
CAT (nmol min^−1^ mL^−1^)	558.3 ± 74.10 ^a^	584.8 ± 53.80 ^a^	613.6 ± 28.24 ^ab^	620.5 ± 64.15 ^ab^	632.6 ± 53.29 ^ab^	754.8 ± 27.27 ^bc^	797.3 ± 30.99 ^c^	0.037
GSH (µM)	0.9 ± 0.10 ^a^	0.9 ± 0.02 ^a^	1.0 ± 0.19 ^a^	1.1 ± 0.09 ^a^	1.2 ± 0.12 ^a^	1.8 ± 0.19 ^b^	1.7 ± 0.21 ^b^	0.002

Values are means ± SE (n = 3). Values with different superscript letters within a row indicate significant differences (*p* < 0.05), whereas values in the same row without superscript letters are not significantly different. Abbreviations: SOD, superoxide dismutase; CAT, catalase; GSH, glutathione.

**Table 8:** Immune parameters of juvenile black rockfish fed experimental diets containing different levels of blood orange juice by-product extract (BJBE) for 8 weeks.

Parameters	Experimental Diets							*p* Value
	BJBE0	BJBE0.1	BJBE0.2	BJBE0.3	BJBE0.5	BJBE0.7	BJBE1	
Lysozyme activity (U mL^−1^)	2.0 ± 0.24 ^a^	2.4 ± 0.34 ^ab^	2.5 ± 0.39 ^abc^	2.6 ± 0.36 ^abc^	2.8 ± 0.11 ^abc^	3.1 ± 0.10 ^bc^	3.3 ± 0.20 ^c^	0.068
IgM (mg mL^−1^)	181.1 ± 11.28 ^a^	193.3 ± 31.67 ^ab^	197.8 ± 34.86 ^ab^	205.0 ± 7.88 ^ab^	223.9 ± 11.88 ^abc^	276.1 ± 34.03 ^bc^	297.2 ± 28.91 ^c^	0.040
IL-1 (pg mL^−1^)	209.6 ± 21.13	181.9 ± 8.01	153.4 ± 23.12	177.8 ± 39.07	149.3 ± 23.81	184.9 ± 8.36	154.8 ± 18.03	0.511

Values are means ± SE (n = 3). Values with different superscript letters within a row indicate significant differences (*p* < 0.05), whereas values in the same row without superscript letters are not significantly different. Abbreviations: IgM, immunoglobulin M; IL-1, interleukin-1.

## Data Availability

The original contributions presented in this study are included in the article. Further inquiries can be directed to the corresponding author.
